# Enterovirus 71 Maternal Antibodies in Infants, Taiwan

**DOI:** 10.3201/1504.081550

**Published:** 2009-04

**Authors:** Shu-Ting Luo, Pai-Shan Chiang, An-Shine Chao, Guan-Yuan Liou, Reyin Lin, Tzou-Yien Lin, Min-Shi Lee

**Affiliations:** National Health Research Institutes, Zhunan Miaoli, Taiwan (S.-T. Luo, P.-S. Chiang, G.-Y. Liou, M.-S. Lee); Chang Gung Memorial Hospital, Linkou, Taiwan (A.-S. Chao, R. Lin, T.-Y. Lin)

**Keywords:** Enterovirus 71, maternal antibody, seroepidemiology, cohort study, dispatch

## Abstract

Enterovirus 71 (EV71) causes life-threatening disease outbreaks in young children in Asia. This cohort study was conducted to understand the dynamics of maternal EV71 antibodies in Taiwanese young infants. Approximately 50% of neonates had detectable EV71 neutralizing antibodies, which declined to almost undetectable levels by 6 months of age.

Transplacental maternal antibodies protect young infants from infectious diseases. On the other hand, maternal antibodies in young infants may impede vaccine effectiveness and confound interpretation of vaccine-induced immune responses. Thus, a need exists to understand the dynamics of pathogen-specific maternal antibodies in young infants (*1*–*3*).

Enterovirus 71 (EV71) was first isolated in California, USA, in 1969. Since then, EV71 has been isolated globally and causes life-threatening outbreaks in young children in Asia (*4*–*10*). National surveillance data and epidemiologic studies show that infants have an increased risk of severe EV71 infections in Taiwan (*6*–*11*). Consequently, vaccine development for EV71 in Taiwan should target infants. This cohort study was conducted to understand the dynamics of EV71-specific maternal antibodies in young infants in Taiwan.

## The Study

Seropositive rates of EV71 neutralizing antibodies in preschool children have been found to be higher in rural areas than in urban areas in Taiwan (*11*). We chose Chang Gung Memorial Hospital (CGMH) as a study site because it has large obstetric and pediatric populations and serves residents from rural and urban areas in northern Taiwan (*7*). Pregnant women having prenatal examinations at CGMH were invited to participate in the study. Serum samples were obtained from participating pregnant women and their children to measure EV71 neutralizing antibody titers immediately before delivery for pregnant women; at birth for neonates (cord blood); and at 6, 12, 24, 36, and 48 months of age for infants. Institutional review board approvals were obtained from CGMH and from the National Health Research Institutes, according to the Helsinki Declaration. Informed consent was obtained from all mothers of participating infants. This report addresses the dynamics of EV71 maternal antibodies in young infants by 6 months of age.

Laboratory methods for measuring EV71 serum neutralizing antibody titers followed standard protocols (*7*) and used a local strain (TW/E59/2002 [B4 genotype]) and rhabdomyosarcoma cells. Serial serum samples obtained from each pregnant woman and her infant were tested in the same run to reduce assay variations. The starting dilution was 1:8, and the cutoff level for seropositivity was 8. Undetectable titer was assigned a level of 2 for calculation of geometric mean titer (GMT). For determining serostatus (positive or negative), serum samples were tested only at 1:8.

Under the assumption that levels of maternal antibodies decline exponentially and constantly, this study used paired serum samples collected at birth and at 6 months of age to estimate the biological half-life that represents an overall half-life and that is crucial for interpreting antibody responses in young infants. Longitudinal and cross-sectional methods of data analysis were used to estimate the biological half-life of pathogen-specific maternal antibodies (*1*).

Obtaining monthly serum samples from young infants to measure seroprevalence of maternal EV71-specific antibodies is unrealistic. Alternatively, the seroprevalence can be predicted mathematically. As has been shown in other viral pathogens, maternal antibody titers are assumed to follow a normal distribution after natural logarithm transformation and to experience a constant exponential decay over time after an infant’s birth (*1*,*12*). If we assume normal distribution, 4 parameters (initial GMT at age *i*, SD of the distribution of antibody titers, decay rates of antibody titers, and cut-off for seropositivity) are crucial for estimating the seroprevalence in different ages (*12*).

Neutralization antibody titers were log-transformed to calculate the GMT and 95% confidence intervals. Statistical association between 2 nominal or ordinary variables was tested by using the χ^2^ test, McNemar test, Fisher exact test, or Mantel-Haenszel χ^2^ test, as appropriate. All statistical analyses were performed using Microsoft Excel (Microsoft, Redmond, WA, USA) or SAS software (SAS Institute, Cary, NC, USA).

**Figure 1:**
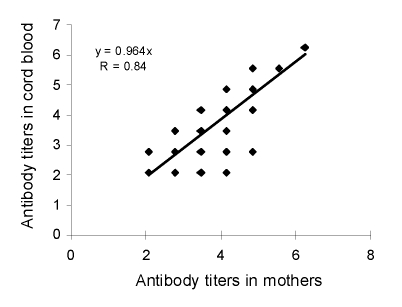
Scatter plot and correlation of enterovirus 71 neutralizing antibody titers (natural logarithm transformation) in 154 pairs of serum samples collected from seropositive neonates and their mothers, Taiwan. Values on the axes are logarithmic.

Serum samples from 459 pregnant women and their neonates were obtained from June 2006 to June 2008 and tested for EV71 neutralizing antibody serostatus. Seropositive rates of EV71 neutralizing antibodies in these pregnant women and their neonates were 63% and 51%, respectively. Seroprevalence in mothers was strongly associated with seroprevalence in their neonates, and neonates born to seronegative mothers were all seronegative (p<0.01, by McNemar test). In addition, the EV71 antibody titers in seropositive neonates were highly correlated with the EV7 antibody titers in their mothers (R = 0.84, p<0.01) (Figure 1).

From June 2006 through June 2008, a total of 309 neonates completed follow-up and blood collection at 6 months of age. EV71 neutralizing antibody titers in the serial serum samples from these 309 families were quantified. The seropositive rates of EV71 neutralizing antibodies in the family cohorts were 65% in the mothers, 50% in the neonates, and 1% in the 6-month-old infants, respectively (Table 1). Only 4 infants 6 months of age had detectable EV71 antibody titers; 1 showed seroconversion on the basis of antibody titers measured at birth (antibody titer <8) and at 6 months of age (antibody titer 512). This seroconverted infant did not develop any enterovirus-related symptoms (e.g., hand, foot, and mouth disease or herpangina). Of the 154 seropositive neonates, 3 remained seropositive and 151 became seronegative at 6 months of age (Table 2). In the 3 seropositive 6-month-old infants, the biological half-life of EV71 neutralizing antibodies was calculated as 39 and 42 days by using cross-sectional and longitudinal analyses, respectively. In the 151 seronegative 6-month-old infants, the biological half-life of EV71 neutralizing antibody was calculated as 53 and 60 days by using cross-sectional and longitudinal analysis, respectively (Table 2).

**Table 1:** Distribution of enterovirus 71 neutralizing antibody titers in pregnant women, neonates, and 6-month-old infants in a cohort study, Taiwan

Antibody titer	No. (%) pregnant women, n = 307	No. (%) neonates, n = 309*	No. (%) 6-month-old infants, n = 309*
<8	107 (34.9)	155 (50.2)	305 (99.0)
8	79 (25.7)	44 (14.2)	3 (0.97)
16	48 (15.6)	48 (15.5)	0
32	34 (11.1)	24 (7.8)	0
64	24 (7.8)	29 (9.4)	0
128	13 (4.2)	6 (1.9)	0
256	1 (0.3)	3 (1.0)	0
512	1 (0.3)	0	1 (0.32)

*Two mothers delivered twins.

**Table 2:** Biological half-life of maternal enterovirus 71 neutralizing antibodies in 154 seropositive neonates, determined by cross-sectional and longitudinal analyses, Taiwan*

Antibody titers at 6 mo of age	No.	GMT at birth (SD†)	GMT at 6 mo	Cross-sectional mean half-life‡	Longitudinal mean half-life§
Detectable	3	203	8	39	42
Undetectable	151	21	2	53	60
Total	154	22 (0.91)	2	53	60

*GMT, geometric mean titer. †After natural logarithm transformation. ‡Calculated on the basis of the GMT at birth and 6 months of age. For comparison, half-life is reported in days, assuming that1 month is equal to 30 days. §Calculated on the basis of paired antibody titers in each individual.

**Figure 2:**
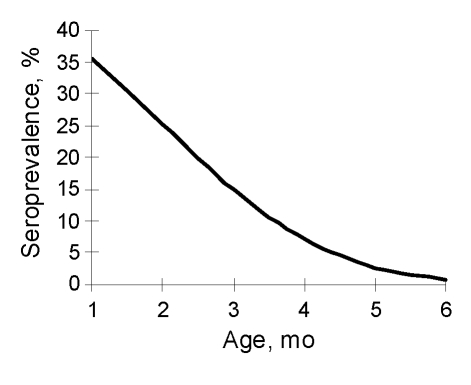
Predicted seroprevalences of maternal enterovirus 71 (EV71) neutralizing antibodies (antibody titer >8) in infants <6 months of age, Taiwan. Predictions are based on assumptions that 1) the seroprevalence in neonates is 50%, 2) the geometric mean titer (SD after natural logarithm transformation) in the seropositive neonates is 22 (0.91), and 3) the half-life of maternal EV71 neutralizing antibodies in young infants is 42 days.

Seroprevalence rates were predicted by making the assumptions that the seropositive rate of EV71 neutralizing antibodies at birth is 50%, that the GMT of these seropositive neonates is 21.8 (SD 0.91), and that the half-life of maternal EV71 neutralizing antibodies is 42 days (1.4 months). Seroprevalence rates of EV71 neutralizing antibodies during each of the first 6 months of age were predicted to be 35%, 25%, 14%, 7%, 3%, and 1%, respectively (Figure 2). The predicted rate at 6 months of age was consistent with the observed seroprevalence at 6 months of age in our study population.

## Conclusions

We found that ≈50–60% of pregnant women had serum EV71 neutralizing antibodies. These maternal EV71 neutralizing antibodies declined to undetectable levels in 99% of the 6-month-old infants. Two cross-sectional studies found similar seropositive rates in 30–49-year-old adults and in 6–11-month-old infants before the 1998 epidemic in Taiwan (*10*,*11*). In Singapore, a cross-sectional study found that 44% of 70 neonates had EV71 neutralizing antibodies in cord blood samples, but none of 52 infants 1–11 months old was seropositive (*13*).

Studies estimating the biologic half-life of EV71 maternal antibodies appear to be new. Theoretically, longitudinal analysis is more reliable and has narrower confidence intervals than cross-sectional analysis (*1*). Although only 3 infants had detectable antibody titers at 6 months of age, our study estimates the biological half-life of EV71 maternal antibodies to be 42 days, similar to the half-lives of antibodies to other pathogens calculated by using longitudinal analysis (*1*; M.-S. Lee, unpub. data).

Our prospective serologic study in northern Taiwan showed no seroconversion in young infants during 2007 and only 1 seroconversion during the first half of 2008. Based on the national enterovirus surveillance system, EV71 isolations were very low in 2006 and 2007 (*14*,*15*), findings consistent with our study.

A national program for developing EV71 vaccines was initiated in Taiwan in 2007. To improve vaccine development, the target population (those at high risk) needs to be identified. Several studies have shown that infants 6–11 months of age in Taiwan have the highest risk for severe EV71 infection and for death caused by this infection (*8*–*10*). Our serologic study found that 99% of 6-month-old infants have undetectable maternal EV71 neutralizing antibodies. Consequently, EV71 vaccines being developed in Taiwan should target infants <6 months of age.
